# Oxidation-Induced Mixed Disulfide and Cataract Formation: A Review

**DOI:** 10.3390/antiox14040425

**Published:** 2025-04-01

**Authors:** Marjorie F. Lou, Robert C. Augusteyn

**Affiliations:** 1 School of Veterinary Medicine and Biomedical Sciences, University of Nebraska-Lincoln, Lincoln, NE 68583, USA; 2 School of Pharmacy, University of North Texas Health Science Center, Fort Worth, TX 76107, USA; 3 School of Optometry and Vision Science, University of New South Wales, Kensington, Sydney, NSW 2052, Australia; raugustn@bigpond.net.au

**Keywords:** oxidation, senile cataract, mechanism of cataract formation, glutathione, lens crystallin proteins, protein aggregation, lens transparency, protein–thiol mixed disulfides, redox regulation, thioltransferase

## Abstract

The major function of eye lens is to transmit light onto retina and form an image. This relies on the crystallin proteins, which are tightly packed to achieve a high refractive index and transparency. The proteins are protected and maintained in a reduced state with intrinsic antioxidants, such as glutathione (GSH), and redox-regulating enzyme systems, such as thioltransferase to maintain the SH/-S-S-balance. When the protective systems are impaired or reduced due to aging, oxidative stress can lead to SH/S-S imbalance, protein modification, protein–protein aggregation and loss of transparency (cataract). Oxidative stress is considered the major culprit in senile cataract formation since cataractous lenses are typically low in GSH content and have elevated levels of GS-protein mixed disulfide (PSSG). This review will examine PSSG accumulation with age and cataracts and explore the possible role of oxidants such as H_2_O_2_. It will also discuss the hypothesis that PSSG formation is not simply a consequence of cataract formation but can trigger the cascade of events leading to loss of lens transparency. The hypothesis is supported by the findings that cataract formation is more rapid with increasing age due to weaker TTase activity and, in animal model systems, when the TTase gene is deleted.

## 1. Introduction

The eye lens is a unique organ with unique properties. It grows throughout life, but because it is surrounded by an elastic capsule, it is not able to shed old cells. Instead, these cells are gradually compacted into the center of the lens as new cells are laid down over them. None of the cell proteins are lost. Thus, the lens retains a record of its own development, growth, and aging. The cells in the absolute center of the lens are older than the chronological age of the subject as they were produced during prenatal growth. Unlike other species, growth in the human lens is biphasic, comprising a rapid prenatal phase, which finishes in the first postnatal year, followed by a constant increase for the rest of life [1]. Different proteins are produced during these two growth phases [2]. As a result, the human lens consists of two distinct regions with different properties—the central nucleus derived from the prenatal growth phase and the outer cortex, which is laid down throughout postnatal life [1]. The two regions are separated by a barrier that restricts the diffusion of small molecules such as water and antioxidant glutathione [3].

The function of the lens is to focus and filter light on its way to the retina. It does this with a refractive index gradient that is generated through the compaction of the cells to produce high concentrations of the lens proteins (α-, β- and γ-crystallins). Crystallin concentrations range from around 150 mg/ml in the outer cortex to almost 400 mg/mL in the center [1]. Close packing of the proteins (short-range, liquid-like order [4]) ensures the tissue is transparent. Disruption of this packing results in lens opacity, i.e., cataract.

The human crystallins have different thiol (SH) contents, arising from cysteine, ranging from 2 residues/mole in some α- and β crystallin polypeptides up to 7 in some of the γ-crystallins. Around half of these are on the surface of the proteins [5,6], easily accessible for modification such as the formation of mixed disulfides with glutathione (GSH) or cysteine (CSH). With the exception of γ_S_-, the γ-crystallins are only produced in the prenatal growth phase [2]. Consequently, the lens nuclear region has a higher average protein SH (PSH) content than the cortex. In conjunction with reduced antioxidant capacity, this makes the nuclear proteins more susceptible to oxidative damage. Furthermore, because there is no turnover of cell contents at any time, lens proteins accumulate a number of post-translational modifications with increasing age. These include polypeptide truncation, deamidation, racemization, and crosslinking [7] which alter their conformation and interactions, making the lens more susceptible to further damage and opacification, i.e., senile cataract, after about age 60.

## 2. Senile Cataract

Cataracts are opacities in the eye lens, which interfere with the passage of light to the retina. Most are associated with increasing age and may be classified as cortical or nuclear or mixed, indicative of their location in the lens.

Cortical cataracts (Type I cataracts [8]) manifest as water clefts (spokes), which span from the capsule towards the nucleus. They appear to be due to disruption of the salt balance of the cortex because of failure of the ion pumps and/or capsular damage. The factors responsible have not been identified but diabetic stress is a common cause [9,10] and whole lens culture studies [11,12] suggest oxidative stress may also be. Nuclear cataracts are characterized by increasing nuclear color ranging from yellow to dark brown (Types II to IV [8]) and even to black (Type V) in extreme cases found in regions where access to ophthalmological services is limited. The color development is accompanied by decreases in the solubility of the nuclear proteins and substantial oxidative damage [13,14,15].

It has been known and accepted for well over a century that oxidative stress, which damages DNA, proteins, and membranes in the lens, plays a role in the development of senile nuclear cataracts [16,17,18]. However, uncovering the mechanism involved has been a slow process, and even today, the sequence of events has not been identified. One of the major changes observed is the increasing loss of protein thiols with increasing lens color [13]. This is due to the formation of protein–protein disulfides (PSSP) and mixed disulfides (PSSX) between proteins and small thiol molecules, predominantly glutathione (GSH), the major antioxidant in the lens. As will be discussed in this review, the formation of mixed disulfides can probably be attributed to the failure of the antioxidant and dethiolating mechanisms.

Views differ on the significance of the elevation in mixed disulfides. One view is that it is simply a consequence of the increased oxidant levels in the lens and plays no direct role in the formation of the cataract [19]. Another view is that mixed disulfide formation causes cataracts by inducing conformational changes in the lens proteins, which make them more susceptible to further damage [20]. It is possible that both participate in the process of cataract formation. In this review, we will analyze and explore each viewpoint.

## 3. Glutathione and Protein–Thiol Mixed Disulfides in the Lens

The possible presence of protein-bound thiols in the lens was first suggested by Hermann and Moses in 1945 but no data were provided [21]. Kinoshita in his 1964 review [22] discussed the loss of GSH in ionizing and galactose cataracts and suggested that mixed disulfides might be formed, impairing metabolic functions. The observation of decreased levels of GSH in human cataractous lenses [23,24,25] led to the expectation that mixed disulfides could be generated during the development of senile cataracts. Mostafapour and Reddy [26] showed that mixed disulfides could be formed with bovine lens proteins through disulfide exchange with oxidized GSH. The amounts formed were in proportion to the thiol content of the individual crystallins.

The first demonstration of mixed disulfides in the human lens was by Harding in 1970 [27]. He measured free and protein-bound GSH in normal and early cataractous lenses and reported that the concentration of free GSH decreased from around 4 mM to 2 mM between birth and age 70. Protein-bound thiol (PBSH) was 0.46 μmole/gm lens wet weight in normal and mixed Type I cataractous lenses. With the early development of nuclear color (Type II cataract), free GSH decreased by a further 50% and PBSH increased to 0.82 μmole/gm lens wet weight. These PBSH levels equate to about 30 and 50 mmoles/mole protein, respectively. The various estimates of mixed disulfides in the human lens are summarized in Table 1 and Table 2.

Srivastava and Beutler [28] found free thiol levels of 3.5–4.5 mM and bound thiols of 5–90 mmoles/mole protein in normal human lenses aged over 65 years. Mixed human cataracts of similar ages were reported to contain 0–1.5 mM free GSH and 94–300 mmoles of PBSH/mole protein. The PBSH levels are up to six times greater than those found in other laboratories (Table 1). Reddy and Han [29] detected 56 and 95 mmoles/mole protein of mixed disulfides in two normal human lenses.

It is difficult to compare the bound thiol levels reported by different laboratories because of the diverse ways in which data were presented: μmoles/gm lens wet weight, μmoles/gm lens protein, nmoles/μmole lens protein, μmoles/lens. Therefore, all data discussed in this review were converted to mmoles bound thiol/mole protein using the average lens protein contents determined by Augusteyn (30–32% for the cortex and 35–36% for the nucleus of the human lens [30]) and 20,000 as the molecular weight of a crystallin polypeptide. Free thiol concentrations were converted to molarities after adjusting for the partial specific volume of proteins (0.74 [31]). The adjusted data are summarized in Table 1 and Table 2.

All of these early studies were performed with small numbers of lenses and/or mixed cataracts of unknown etiology. Truscott and Augusteyn [13] undertook a more comprehensive approach to examine the relationships between PSH, NPSH, and PBSH in age-related nuclear (ARN) cataractous human lenses. They presented detailed data on the levels of these in each of the separated cortex and nucleus from 4 normal and 34 cataractous lenses and showed that, with increasing lens nuclear color (Types II–IV cataract), nuclear PSH and NPSH levels decreased to under 10% of those in the normal lens while PBSH (of which over 95% was GSH) increased. The changes were the greatest in the nucleus where PBSH increased from 4.4 to 48 mmoles/mole protein compared to the cortical PBSH, which increased from 3.5 to 26 mmoles/mole protein. PSH decreased from 1.8 moles/mole protein to less than 0.05 M/mole protein in the nucleus and from 1.9 to ~0.5 moles/mole protein in the cortex. These changes were accompanied by extensive (45%) oxidation of methionine and large increases of water-insoluble (urea-soluble) and disulfide-linked (PSSP) yellow proteins [13,14,15], consistent with the involvement of oxidation in nuclear cataract formation.

The observations of Truscott and Augusteyn triggered a series of studies by Lou and colleagues. Previous estimations of protein-bound thiols were based on the use of colorimetric methods for the quantification of thiol levels after borohydride reduction to cleave the disulfide bonds. There would have been a risk of re-oxidation of the released thiols during the assay. To overcome this risk, Lou et al. [32] used performic acid oxidation to release the thiols from PBSH. The resultant sulfonic acid products were stable and very small amounts (2% of one human lens and 20% of one rat lens) could be reliably quantified using anion exchange chromatography.

Initially, this method was used to assess mixed disulfides in cultured rat and monkey lenses. They found levels ranging from 12 to 41 mmoles/mole protein for GSH mixed disulfides (PSSG). They also found previously undetected protein-bound cysteine disulfides (PSSC) at around seven times higher. Exposure of the rat lenses to H_2_O_2_ increased PSSG but did not affect PSSC [32]. In a later study, Lou et al. [33] examined the redox status of human lenses. In agreement with the previous reports [13,26], they found the PSSG content in pigmented lenses to be about double that of normal lenses.

Lou and Dickerson also measured PSSG and PSSC in normal human, monkey, dog, rat, squirrel, and emu lenses [12]. After allowing for the differences in the lens protein contents in the different species lenses [30], the total mixed disulfide contents ranged from 4 to 20 mmoles/mole protein. Bound cysteine accounted for less than half of all species except for the rat where it represented >80% of the total mixed disulfide.

### 3.1. Mixed Disulfides in the Normal Human Lens with Age

The concentration of GSH in the lens decreases with age while GSSG increases [12,34,35]. Lou and Dickerson [12] determined free GSH and mixed disulfide levels in fifty-nine normal human lenses aged between 3 months and 88 years. In the first decade of life, the concentration of GSH averages 4–5 mM in the lens cortex and 3.5–4.5 mM in the nucleus. The levels decrease with age to around 3 and 1.5 mM, respectively, by age 88. At the same time, protein mixed disulfides increase linearly from 3.3 to 24.4 mmoles/mole protein in the cortex and 4.3 to 40 mmoles/mole protein in the nucleus. The upper levels correspond to around 1 and 1.5%, respectively, of the total PSH content of the lens proteins. Similar, low amounts of mixed disulfide were observed in earlier studies (Table 1). A comparison of the Lou and Dickerson PSSG values with the GSSG data from Rathbun and Murray [34] suggests that PSSG increases linearly about five times faster than GSSG.

Around 33% of the free thiol decreases in the aging lens can be attributed to the formation of GSH–protein disulfides [12] and up to 7% is present as GSSG [34], but the fate of the remaining 60% is not known. Both GSH and GSSG may have leaked [35] or been transported [36,37] out from the lens for the maintenance of surrounding tissues. In addition, GSH synthesis decreases with age [34,37]. As noted by Truscott [38], the decrease in GSH observed with age does not appear to be detrimental, with no evidence of protein damage such as the oxidation of methionine [14]. Lenses with nuclear GSH of 1.4 mM and PSSG of 30 mmoles/mole protein appear normal [39]. It has been suggested that cataracts will result when the GSH concentration drops below 1 mM [40].

**Table 1:** Glutathione–protein mixed disulfides in human lenses.

	Normal Lens				Cataractous Lens			
	Age	Cortex	Nucleus	Whole	Type	Cortex	Nucleus	Whole
Lou and Dickerson [12]	19–21			16				
	<10	3	4	5				
	11–80	3–24	4–60	40				
Truscott and Augusteyn [13]	45–62	3.5	4.4		I	10	23	
					II	23	49	
					III	25	44	
					IV	26	48	
Lou, Huang and Zigler [33]	na		16		Mixed			44
	na		18		Mixed			32
	61			25	IV			49
	98			16	IV			53
Lou, Dickerson, Tung, Wolfe, Chylack [39]	87–89		14		I		18	
					II		25	
					III		52	
					IV		57	

Data are expressed as mmoles of bound thiol/mole protein. Values in the table were calculated from the literature data using the average protein content of the lens (32%, Augusteyn [30]) and 20,000 as the molecular weight of crystallin polypeptides.

**Table 2:** Cysteine–protein mixed disulfides in human lenses.

	Normal Lens				Cataractous Lens			
	Age	Cortex	Nucleus	Whole	Type	Cortex	Nucleus	Whole
Lou and Dickerson [12]	19–21			2.5				
	<10	1.1	1.3	1.1				
	11–80	1.4–5.6	1.8–9.4	6.3				
Lou, Huang and Zigler [33]	na			4.6	Mixed			11.1
	61			3.2	IV			58
	98			8.5	IV			26
Lou, Dickerson, Tung, Wolfe, Chylack [39]	87–89		5		I		8	
					II		8.8	
					III		17.5	
					IV		25.6	

Data are expressed as mmoles of bound thiol/mole protein. Values in the table were calculated from the literature data using the average protein content of the lens (32%, Augusteyn [30]) and 20,000 as the molecular weight of crystallin polypeptides.

Similar age changes were observed with cysteine (Table 2). The concentration of free cysteine in the human lens nucleus decreases from around 0.18 to 0.06 mM and PSSC increases linearly from 1 to around 10 mmoles/mole protein, generally about twice that of the cortex [41]. Essentially all of the cysteine decrease is due to an increase in PSSC. In a later study, Dickerson and Lou [42] found a third PBSH compound, γ-glutamylcysteine (PSSGC), at about the same level as the PSSC in human lenses aged over 60 years. They speculated that γ-glutamylcysteine, an intermediate in GSH synthesis, accumulated because of reduced biosynthesis in the aging lens.

**Figure 1:**
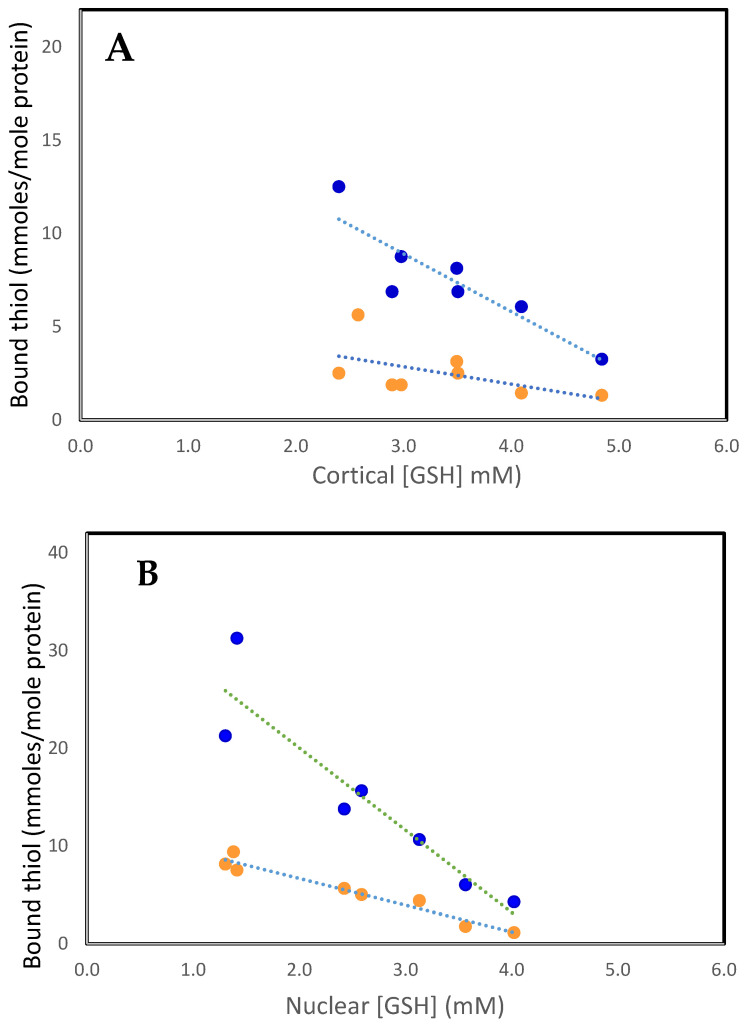
The relationship between PSSC (• • •) and PSSG (• • •), with free GSH in the human lens. (**A**). Cortex. PSSC = 5.66–0.94 [GSH], R^2^ = 0.3, *p* < 0.03: PSSG = 18.2–3.09 [GSH], R^2^ = 0.8, *p* < 0.007. (**B**). Nucleus. PSSC = 12.13–2.73 [GSH], R^2^ = 0.95, *p* < 0.0001: PSSG = 36.85–8.42 [GSH], R^2^ = 0.86, *p* < 0.003. The plots were constructed using the data of Lou and Dickerson [12].

As can be seen in Figure 1A,B, the increases in bound GSH (PSSG) and bound cysteine (PSSC) are highly correlated with the decreases in reduced GSH levels in both the cortex and nucleus. Generally, PSSG levels are 4–6 times those of PSSC. This is a surprising observation given that free GSH levels are up to 50-fold higher than those of free Cys [41]. Since GSH (GSH/GSSG, cytosolic redox potential of −220 to −320 mev) is a stronger reductant than Cys (Cys/CSSC, −80 mev), electron transfer would be expected to occur from GSH to CSSC, maintaining Cys in its reduced form and minimizing PSSC formation. Perhaps cysteine, due to its smaller size, is bound at protein sites inaccessible to GSH. Cysteine is an important component of metabolic systems [43], but its function in the lens is not understood, especially in the metabolically inert nucleus. It seems unlikely that it is a general antioxidant such as GSH. Perhaps its function in the cortex is to form mixed disulfides with protein thiols as part of redox regulation of activities or to protect them from oxidative damage. However, PSSC is a poor substrate compared to PSSG for reduction by the redox regulator thioltransferase (glutaredoxin) [44]. Furthermore, the activity of thioltransferase is very low in the lens nucleus. Thus, if PSSC is formed, it would be improbable for the protein to be reduced back to its functional PSH form.

### 3.2. A Possible Function of Mixed Disulfides in the Lens

It has been suggested that the thiolation of protein sulfhydryl groups provides temporary protection against damage for key lens metabolic components during periods of stress [25,45,46,47,48], as has been demonstrated in other tissues [49]. When the stress has abated, the process can be reversed, and normal functions are restored. Benedek et al. [45] showed that the formation of mixed disulfide between bovine γIVa-crystallin and glutathione suppressed the aggregation of the protein. They concluded that the mixed disulfide might prevent the formation of protein–protein disulfide links, thereby slowing cataract development. The rat lens is capable of restoring glutathione levels and decreasing PSSG levels (but not PSSC) when oxidative stresses are removed but only after short-term (24-h) exposure to H_2_O_2_ [50]. This suggests that there may be a mechanism for releasing the bound thiols.

It is reasonable to assume that the lens, like other organs, would have mechanisms to protect it from unwanted post-translational changes that may damage its structural proteins or inactivate key enzymes. In some proteins, a GS- or CS- adduct on exposed SH sites would be the fastest way to protect them initially. However, without a timely de-thiolation of the adduct, the extra size and charge of PSSG or PSSC side chains may cause instability and conformational changes resulting in crosslinking with neighboring molecules to form large aggregates that interfere with lens transparency. If this were the case, a cataractous tissue would be expected to show elevated levels of protein–thiol mixed disulfides.

Srivastava and Beutler [28] reported that glutathione reductase could cleave the mixed disulfide bond, but Latta and Augusteyn [51] showed that the highly purified lens enzyme could not. It is likely that the enzyme preparation used by Srivastava and Beutler contained free GSH, which can cleave the bond [52]. These considerations led Lou and colleagues to explore possible mechanisms for cleaving the mixed disulfide bond. As will be discussed later, they demonstrated that the lens contains a number of dethiolating systems, including thioltransferase, which, with GSH, can reduce PSSG back to PSH.

### 3.3. Mixed Disulfides and Cataracts

With nuclear cataract development, the concentration of free GSH decreases even further than with age [13,22,23,24,26] falling below the suggested 1 mM threshold for cataract formation [40] and continuing to decrease with increasing nuclear color. In the most advanced cataract (dark brown, Type IV [8]), nuclear GSH drops to 0.15 mM [12,39]. Why this occurs is not understood but it may be due to a surge in the level of H_2_O_2_ as a consequence of the precipitous drop (70%) in nuclear glutathione peroxidase and superoxide dismutase activities with the onset of nuclear cataract (Type II lenses) followed by the almost complete loss in Type IV lenses [53].

**Figure 2:**
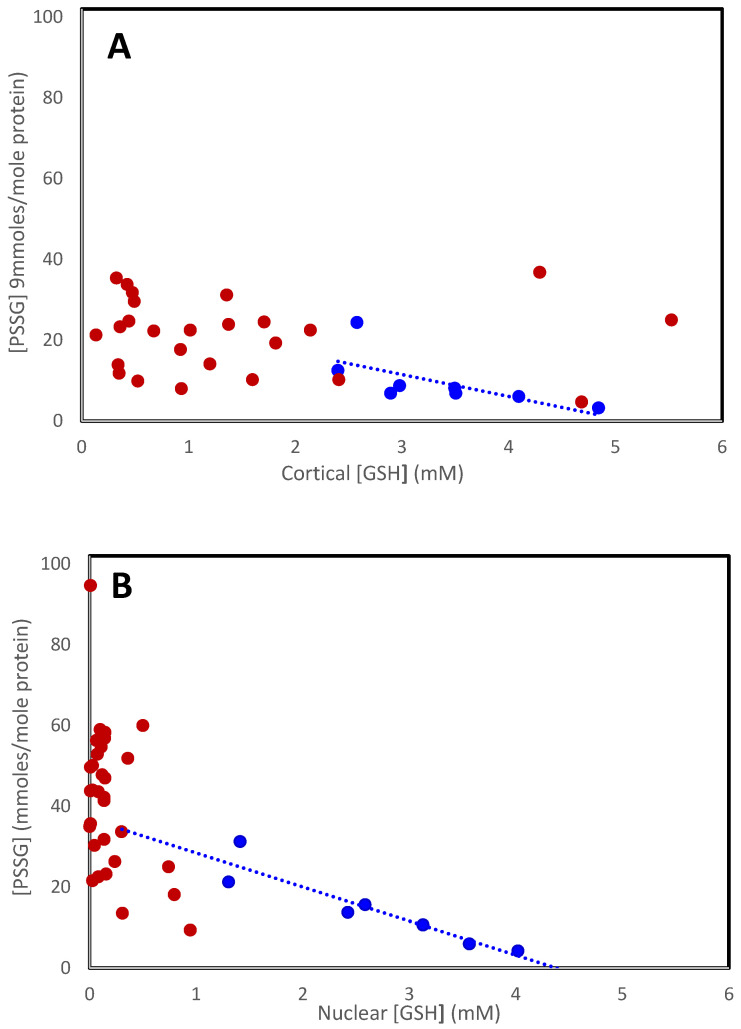
The relationship between PSSG and [GSH] in the cortex (**A**) and nucleus (**B**) in normal (• • •) and cataractous (• • •) lenses. Data were obtained from Lou and Dickerson [12], Truscott and Augusteyn [13], and Zhang et al. [54].

The increase in mixed disulfides observed with age continues with the further decrease in GSH in cataracts. Figure 2 shows the relationship for all lenses examined [13,39]. Mixed disulfide levels in the cataractous cortex (Figure 2A; red symbols) appear to increase in the same way, relative to [GSH], as in normal lenses (blue symbols), reaching a maximum of around 35 mmoles/mole in the Type IV lens. The levels are much more variable in the nucleus (Figure 2B), probably reflecting the use of data from a variety of different and mixed cataracts, with and without nuclear color. As can be seen in Table 1, protein-bound thiols increase with nuclear lens color (Type II–IV). In the Type IV cataractous lens, nucleus PSSG reaches around 60 mmoles/mole, but the combined PSSG + PSSC + PSSGC can reach nearly 100 mmoles/mole [39,42].

The increase in mixed disulfides is not limited to nuclear cataracts. The Lou laboratory performed a detailed study of the thiol status in the nucleus + inner cortex obtained from extracapsular extraction (ECCE) of mature, nuclear, cortical, posterior subcapsular cataracts (PSC), nuclear/PSC, and mixed cataracts [54]. GSH was markedly reduced in all cataract types while PSSG was elevated in all, especially in nuclear and NPSC lenses (20 and 28 mmoles/mole protein, respectively). Interestingly, significant levels of PSSC (up to 12 mmoles/mole protein) were observed in mature, NPSC, and mixed cataracts, suggesting these may involve additional mechanisms.

It has been proposed that the PSSG increase is the trigger for cataract development [20]. Conformational changes due to PSSG formation are proposed to lead to disulfide-linked proteins (PSSP) and then to protein–protein aggregation with eventual cataract formation. This is discussed in the next part of this review. In support of this proposal, it was shown that PSSG formation can induce conformational changes in isolated bovine lens proteins [55,56]. However, because of the nature of these studies, protein–protein disulfide links, insolubilization, and color development, typical of nuclear cataracts, could not be observed.

Support for the trigger hypothesis could also come from a number of studies that have described conformational changes in lens proteins during nuclear cataract formation. Unfortunately, none measured mixed disulfide contents. Changes have been found in the conformation of total human lens proteins (soluble + insoluble) during cataract development [57,58]. These were shown to be restricted to the insoluble proteins, while the proteins remaining soluble were largely unaffected [59]. Minor conformational changes were detected in the isolated soluble proteins from human cataractous lenses following prolonged exposure to H_2_O_2_ [60]. These proteins were first reduced and dialyzed, so no low molecular weight compounds (including thiols and disulfides) from the lens should have been present. This would indicate that the formation of mixed disulfides is not a contributor to nuclear cataract development but rather a consequence. However, the possibility remains that GSH trapped within the proteins played a role in the cataract-like changes.

The highest level of mixed disulfide reached represents only 4–5% of the protein thiol lost. This may represent an initial thiolation response to stress. The majority (>90%) of the protein thiol decrease is through the formation of protein–protein disulfide bonds [6,13]. This only takes place during the development of nuclear color (Type II–IV). However, it may be that the slow age-related accumulation of mixed disulfides in sensitive regions eventually leads to disruption of the protein structure and cataracts.

Evidence in support of the ‘trigger’ hypothesis is presented in the following sections.

## 4. The Nature of the Oxidant Responsible for Cataracts

The thiol losses, mixed disulfide formation, methionine oxidation, and other protein damage in nuclear cataracts [13,14,15] are probably the result of oxidative stress generated by reactive oxygen species (ROS); hydrogen peroxide (H_2_O_2_), superoxide (·O_2_^−^), and hydroxyl radicals (·OH) [19]. These can be produced within the lens by the univalent reduction of oxygen [18] or by external agents such as exposure to near-UV radiation [61,62] or hyperbaric oxygen [63].

The human lens contains several protective mechanisms that can detoxify these oxidants [53], and glutathione is an important component of these [19,64]. With the onset of a nuclear cataract (Type II), the activities decrease precipitously. In particular, glutathione peroxidase decreases by around 70% in the nucleus and over 50% in the cortex, suggesting that there may be a marked reduction in the ability of the lens to detoxify endogenous and exogenous H_2_O_2_. The activity of glutathione reductase also decreases [65] and, together with elevated H_2_O_2,_ could lead to an increase in GSSG and subsequent PSSG formation. As detailed earlier, both disulfides were found in cataractous lenses, especially in the colored nucleus [6,13,27,39]. In addition, the nuclear proteins were found to contain methionine sulfoxide and extensive protein–protein disulfide links [6,13].

To evaluate the hypothesis that H_2_O_2_ was responsible, McNamara and Augusteyn [60] isolated soluble proteins from the nucleus of Type I lenses (no colored nucleus), reduced any disulfides, and, after removal of the reducing agent and released thiols, incubated the proteins for several weeks with different concentrations of H_2_O_2_, up to 1.0 mM. (The proteins were in dialysis bags immersed in baths of buffered H_2_O_2_, which were maintained at the required concentration with daily adjustments). Progressive and concentration-dependent changes, typical of nuclear cataracts, were observed in the properties of the proteins. These included PSH loss, methionine oxidation, covalent crosslinking, increased non-tryptophan fluorescence (conformation change), and extensive insolubilization. These observations provided the first substantial support for the idea that H_2_O_2_ is responsible for age-related nuclear (ARN) cataracts. Consequently, H_2_O_2_ has been most commonly used in cataract model studies.

The source of the oxidant has not been determined. It has been reported that H_2_O_2_ levels in the aqueous humor are elevated in some cataracts, suggesting that penetration of H_2_O_2_ could be responsible for the oxidative damage [66,67]. As will be detailed later in this review, whole lens cultures have been used to explore this possibility in rats [11,20,50] and humans [12]. Exposure of 65-year-old human lenses to 0.5 mM H_2_O_2_ for 36 hours resulted in lens haziness, swelling, and cortical spokes [12] indicative of epithelial damage and alterations in the salt balance of the cortex. As first shown by Fukui [11], this can probably be attributed to inhibition of the cation transport mechanism. At the same time, GSH was lost while PSSG and PSSC increased. The changes were more pronounced in the cortex than in the nucleus.

Similar changes were produced by incubation of bovine lenses with 30 mM H_2_O_2_ [68]. These included wet weight increase, haziness, almost complete loss of free GSH, and formation of PSSG (0.4 moles/mole protein) and PSSC (0.04 moles/mole protein). Of particular interest was the observation that all seven methionine residues in γB crystallin were partly oxidized to the sulfoxide. This had also been found with isolated α-crystallin incubated with 1 mM H_2_O_2_ /FeCl_3_ [69]. These observations provide further support for the involvement of H_2_O_2_ in both cortical and nuclear senile cataract formation. However, the source of the oxidant remains unclear.

While the whole lens culture can reveal the effects of exogenous challenges on the epithelium and cortex, it is unlikely that significant amounts of H_2_O_2_ can survive the cortical defense systems and penetrate the nucleus without causing substantial epithelial and cortical damage on the way. Unlike the nucleus, the cortex still has significant catalase, glutathione peroxidase, and superoxide dismutase activities [53]. Furthermore, as shown by Truscott and colleagues [3], the human lens nucleus is surrounded by a barrier to the diffusion of GSH and water, which may also restrict the entry of peroxide. Thus, whole lens cultures can provide information on the ability of the cortex to withstand oxidative stress, but not the nucleus. Exogenous peroxide may be responsible for cortical cataracts but not nuclear cataracts. On the other hand, some external agents, including hyperbaric oxygen (HBO) and near UV, can affect the lens nucleus in experimental animals [61,62,63].

Giblin et al. [63] found that prolonged exposure of guinea pigs to HBO resulted in nuclear light scattering, with concomitant decreases in protein solubility and protein thiols and increases in PSSG and CSSG. The cortex was largely unaffected. It would appear that the increased oxygen levels in the nucleus (up to 12.5-fold) coupled with metal ions could lead to ROS production.

Exposure of squirrels and guinea pigs to near UV (300–400) for up to a year resulted in loss of lens GSH and increased PSSG and CSSG, with more in the nucleus than in the cortex [61,62]. This has been attributed to the formation of ROS in photosensitized reactions in the nucleus [62]. However, the relevance to human cataracts is unclear since epidemiological studies have shown that exposure to sunlight (near UV) generates cortical cataracts but does not affect the nucleus [70].

## 5. The Possible Role of Mixed Disulfides in Triggering Cataract Formation

**Figure 3:**
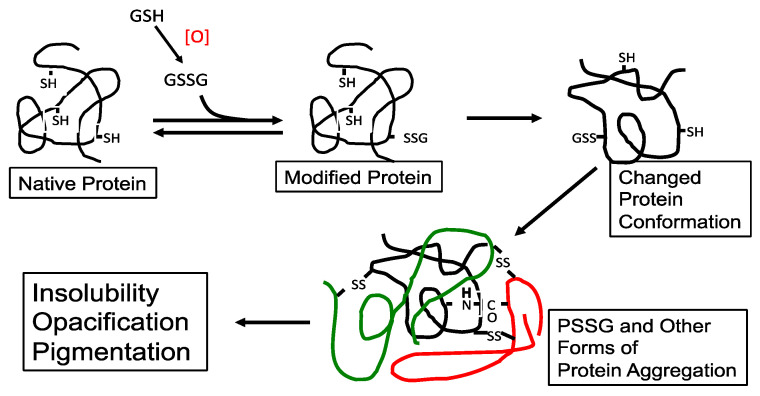
The possible role of protein–thiol mixed disulfide (PSSG) in lens protein aggregation during oxidation-induced cataract formation. An aggregate of three proteins (different colors) is shown. Reprinted from Lou [44] with permission from Pergamon.

It was proposed in the early 90′s [20] that during the early stage of oxidative stress, the formation of protein–thiol mixed disulfides (protein thiolation), in particular GSH adducts (PSSG), can be a triggering point leading to a cascade of protein-S-S-protein (PSSP) formation and other protein modification, with eventual protein–protein aggregation and cataract formation (Figure 3). A similar hypothesis on the possible role of protein–thiol mixed disulfides in oxidative damage to proteins from other tissues was also suggested by Brigelius [71].

The following section of this current review will focus on the evidence showing how protein thiolation can alter protein conformation to trigger the events in cataract formation and how a dethiolating enzyme, thioltransferase (TTase), or glutaredoxin (Grx), can dethiolate PSSG, thus retaining the thiol/disulfide balance in lens proteins and preventing the adverse effects of PSSG on lens function. This topic has, in part, been reviewed previously by Lou [44,72].

## 6. Glutathionylation of Lens Protein Causes Conformational Changes

The possibility that the large side chain and extra charge introduced during the formation of GSH adducts (glutathionylation) may affect the physical properties of the protein has been examined in several laboratories. Liang and Pelletier [55] first reported that PSSG formation of purified γ-crystallin induced conformational changes so that the protein became unstable and more susceptible to proteolysis. A later study by Kono, Sen, and Chakrabarti [56] compared the conformational stability of several γ-crystallins from the bovine lens, using far-UV CD and fluorescence, and confirmed that GS-adducts indeed caused a dramatic reduction in stability.

Hanson et al. [68] provided a more in-depth observation of how glutathionylation could alter lens protein structure by examining the thiol modification sites of γB-crystallin isolated from an intact bovine lens that had been exposed to H_2_O_2_ (30 mM) for 24 h. Of the 7 SH groups in γB-crystallin, it is known [73] that 3 are exposed (Cys-15, Cys-22, Cys-41), 2 are semi-exposed (Cys-18, Cys-109), and 2 are buried (Cys-32, Cys-78), inaccessible to bulky reagents. By using mass spectroscopy, these authors detected PSSG formed first at one of the exposed SH sites between residues 10 and 31, similar to the one glutathionylation site usually found in the non-H_2_O_2_ exposed control lens. With H_2_O_2_ exposure, a second GS-adduct appeared at a partially exposed SH site at Cys-109.

Additionally, mass spectrometric examination did not detect any intramolecular disulfide bond in γB-crystallin from a control lens but an intramolecular -S-S- crosslink was found between the buried -SH site at Cys-78 and Cys-41, and between Cys-78 and peptide 10–31 after the lens was exposed to oxidative stress. These changes suggest that glutathionylation has indeed induced protein conformation change, in agreement with the two previous reports [55,56]. Therefore, the study by Hanson et al. [68] provided very convincing evidence that PSSG formed in the early stage of H_2_O_2_ stress not only induced conformational change of the lens protein but also caused the opening of structure wide enough to allow for buried SH sites to be accessible by an oxidant, causing irreversible damage. More importantly, all these changes were taking place in an intact lens, not in an isolated protein.

The findings in the above three studies provide strong support for the hypothesis of Lou et al. [20] that under oxidative stress conditions, PSSG that was made initially could trigger a cascading event in protein disulfide (PSSP) formation, modifications of other functional groups, and, finally, lead to aggregation between structural proteins. As lens transparency depends on its unperturbed structural proteins in their native state, the aggregates when large enough would obstruct the light transmission causing the lens to lose transparency.

It is important to point out that PSSG formation in lens proteins is a post-translational process and likely occurs randomly. It may also depend on which SH site is being modified and whether certain proteins are more vulnerable to conformational alteration. Furthermore, the amount of PSSG formed may not need to be very high to initiate the cascading effect.

## 7. Model Studies on the Formation of PSSG in Lens Proteins During Oxidative Stress

Animal lenses were first used to establish a model system of the lens organ culture that would be suitable for studying the changes in the human lens. H_2_O_2_ is often the choice of oxidant to induce cataracts in vitro. Thereafter, studies with both rat lens and human lens were carried out as shown below.

### 7.1. Rat Lens Culture: H_2_O_2_ Exposure and Post-Oxidation Recovery

**Figure 4:**
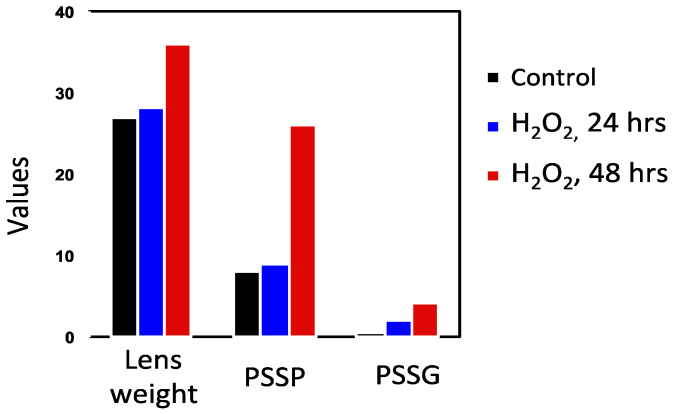
A comparison of the H_2_O_2_-induced onset in PSSG and PSSP formation. Reprinted from Lou [72] with permission from Liebert.

In order to understand the role of protein–thiol mixed disulfide formation in relation to the sequence of events during cataract induction, Lou and associates [50,74] conducted H_2_O_2_ exposure studies using rat lenses for 24, 48, 72, and 96 h to monitor the dynamic changes in morphology and biochemistry. During the first 24 h of exposure, only a patchy opacity was found in the equator. By 48 h, there was a full cortical opacity with lens weight gain, likely due to damage in the membrane pumps. Beyond 72 h, the opacity covered the entire lens. GSH loss and PSSG increase occurred within 2 h and continued so with prolonged H_2_O_2_ exposure. Protein thiolation was first found in the water-soluble proteins (WS), 24 h ahead of the water-insoluble proteins (WI). The formation of PSSC was much slower than that of PSSG, at least 24 h or longer in both the WS and WI fractions. This chronic oxidative stress model also revealed a profound change in the protein profile. Protein–protein disulfide (PSSP) aggregates appeared along with an increased WI-to-WS ratio after 48 h (Figure 4), well correlated with the morphological alteration [50].

Close examination of the mixed disulfide formation in the cortical and nuclear regions showed that, in general, PSSG formation was far greater than that of PSSC. After 48–72 h H_2_O_2_ exposure, PSSG was higher in the cortex than that in the nucleus, while the opposite was true for PSSC, which was elevated more in the nucleus and predominantly in the WI proteins [74].

**Figure 5:**
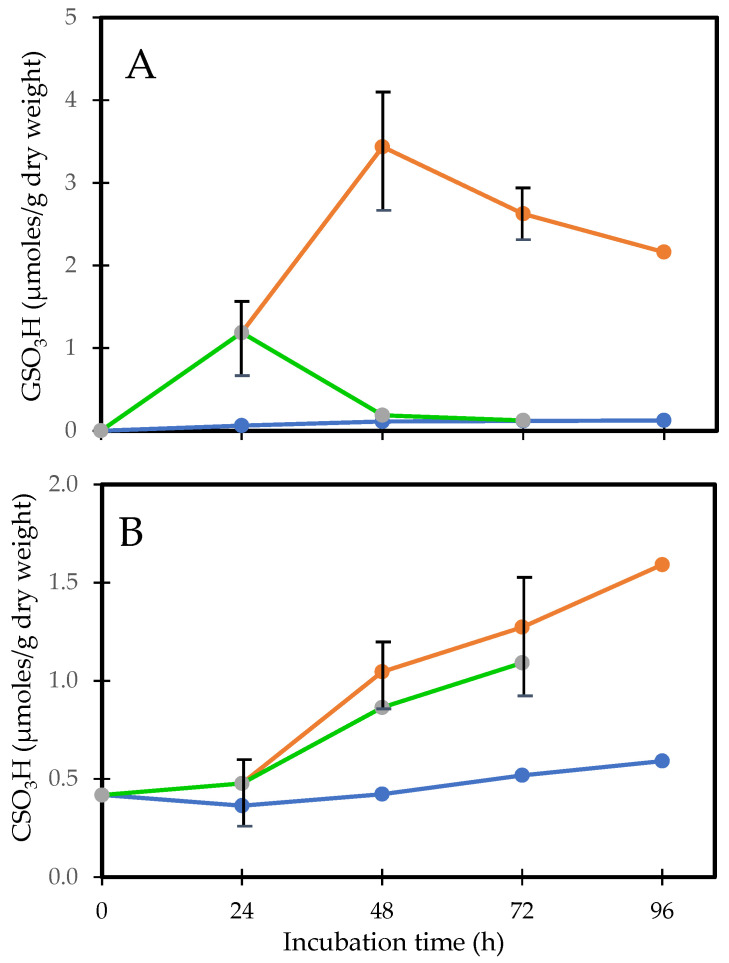
H_2_O_2_-induced protein thiolation in rat lens organ culture and its reversal upon oxidant removal. (**A**). Recovery of PSSG (GSO_3_H), (**B**). Recovery of PSSC (CSO_3_H). Five lenses of the same group were pooled and used for the analysis. (•-•-•) control, (•-•-•) H_2_O_2_, (•-•-•) recovery. Plots from Cui and Lou [50] were redrawn and reformatted.

This study provides evidence for the possible association of protein thiolation with cataract formation. First, PSSG formation is the earliest change (Figure 5A), which is observed within a few hours on the 1st day during oxidative stress, before PSSP formation or protein aggregation (on the 2nd day), followed by lens opacity (3rd day). Thus, the sequence of events aligns with the hypothesis of Lou et al. [20]. Second, it is known from the distribution of thiolated proteins in normal or cataractous rat and human lenses that PSSC are predominantly found in the nuclear region [12,75]. In this model, the PSSC level was unchanged during the first 24 h when the PSSG level was elevated extensively; however, it suddenly increased by 48 h (Figure 5B), suggesting that despite the antioxidants in the cortex, prolonged H_2_O_2_ exposure allowed some of the H_2_O_2_ molecules to reach to the nucleus causing oxidized cysteine (CSSC) to form CS- adducts to some of the nuclear proteins in that region.

The above study was also designed to investigate what effect placing the H_2_O_2_ pre-exposed lenses into an H_2_O_2_-free medium with continual incubation would produce. This led to interesting findings that, within 24 h, PSSG returned to its basal level, PSSP accumulation was also decreased and the H_2_O_2_ pre-exposed lenses resumed nearly the same transparency status as the normal control lens while retaining most of the weight gain [50]. However, PSSC recovery appeared to be slower [50,74], and more so in the nucleus [75]. These findings suggest that the lens may have an intrinsic system for regulating thiol/disulfide exchange and is more effective in regulating PSSG than PSSC. This recovery phenomenon for PSSG may only be achievable for a short-term H_2_O_2_-exposed lens (<24 h). Beyond 24 h, the lens appeared to lose this capability; likely the intrinsic repair or recovery system itself might have been damaged from the prolonged oxidative stress.

### 7.2. Human Lens Culture Studies: H_2_O_2_ Exposure and Post-Oxidation Recovery

**Figure 6:**
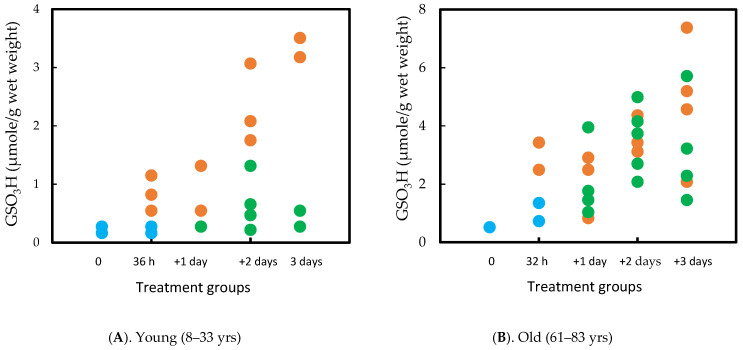
Comparison of the PSSG (measured as GSO_3_H) recovery efficiency between young (**A**) and old (**B**) human lenses. The lenses were exposed to H_2_O_2_ for 32–36 h and then examined after 1, 2, and 3 days in H_2_O_2_ free medium. Control (•) H_2_O_2_ (•) Recovery (•). Each data point represents one lens.

A parallel study was carried out using normal human lenses, which required 36 h of H_2_O_2_ exposure to achieve opacity in the cortex. However, the same recovery was observed in lens transparency, GSH/PSSG homeostasis, and protein solubility as in the rat lens. Interestingly, although aging showed similar recovery ability in rat lenses pre-exposed to oxidative stress between 1 m and 23 m [74], age played a more important role in recovery for the human lens. The capacity was quite efficient in the young (8–33 yrs) but not so in the old (61–83 yrs) lenses. In some of the old lenses, PSSG continued to increase in the absence of H_2_O_2_ in the culture medium. It is likely due to the decreased enzymes in thiol/disulfide regulation as described in the later part of this review. The results are summarized in Figure 6A,B [76].

**Figure 7:**
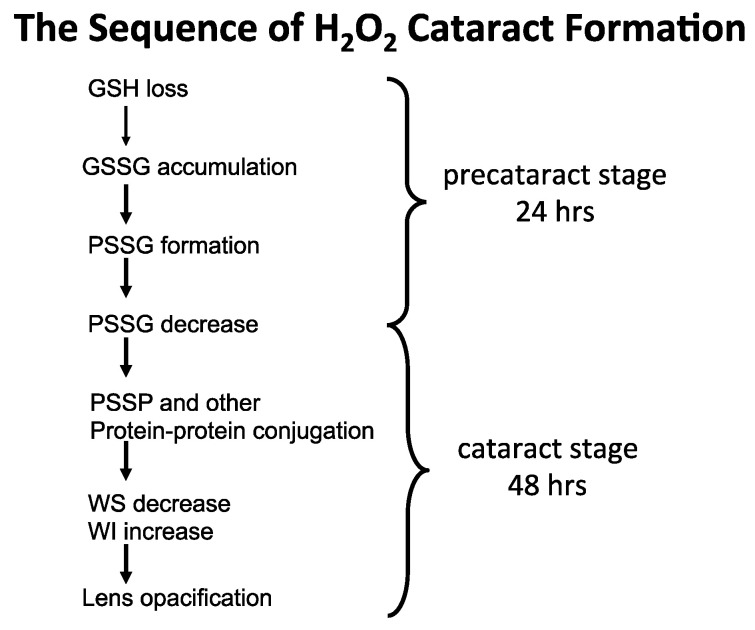
The proposed sequence of H_2_O_2_ cataract formation.

From the above rat and human lens studies, H_2_O_2_-induced cataract formation can be summarized in the following sequence of events, as illustrated in Figure 7.

## 8. The Intrinsic SH/-S-S- Regulating Systems in the Lens

The reversibility of PSSG formation in an oxidative stress pre-exposed lens described above was a surprise finding [50], but it is a perfectly logical scenario, considering a human lens is constantly under oxidative stress throughout life, and yet cataract only begins at an old age. Thus, the lens must possess a repair mechanism to control and protect it from oxidation-induced thiol modification, but if protein thiolation occurs, it must be able to reverse the modification to prevent any malfunction of certain enzymes or proteins within the tissue. In other words, the lens may have a system to regulate redox homeostasis throughout its life span until the oxidative stress is overwhelming or the intrinsic system itself is damaged from the same oxidative stress or weakened during aging. It is then that the lens is prone to cataract formation.

### 8.1. The GSH-Dependent Thioltransferase

The presence of such an intrinsic system was identified in the lens by Raghavachari and Lou [77]. It is a GSH-dependent enzyme called thioltransferase (TTase-1), or glutaredoxin (Grx-1), identical to the TTase found in other tissues, such as the liver [78] and red blood cells [79]. The mechanism of action of TTase is to catalyze thiol–disulfide interchange by reducing protein mixed disulfide to its protein-SH form using GSH as a cofactor. This enzyme favors PSSG over PSSC and is highly oxidation resistant. Lens TTase is mainly found in the epithelium with lesser, but equal, amounts found in the cortex and nucleus. This cytosolic enzyme has similar properties to those of the TTase from other tissues. An isozyme of TTase (TTase-2 or Grx-2) was later found with a specific function in regulating redox balance in the mitochondria of the lens [80]. A more comprehensive review on the cytosolic TTase-1 (Grx1) and mitochondrial TTase-2 (Grx2) authored by Wu and Lou can be found in this special series of “Oxidative Stress in Cataracts: Mechanisms and Therapies”.

### 8.2. The NADPH-Dependent Thioredoxin–Thioredoxin Reductase (TRx-TR) System

Another redox regulating system, the NADPH-dependent thioredoxin and thioredoxin reductase system (TRx/TRx reductase) is found in the lens and works in conjunction with the GSH-dependent TTase system to regulate redox homeostasis in the lens. TRx/TRx reductase has many cellular functions, including converting the thiol-oxidized proteins/enzymes (protein-S-S-protein, or PSSP) to their reduced form (protein-SH, or PSH), thereby restoring protein structural function or enzyme activity.

TRx belongs to the oxidoreductase family with a mass of 12 kDa while TR is a 55 kDa Seleno-protein. TRx has isoforms, TRx-1 and TRx-2, present in the cytosol and mitochondria, respectively. TRx specifically catalyzes the reduction of inter- or intra- protein–protein disulfides, and sulfenic acid at the cysteine moieties of proteins, thus regulating the activities of these modified proteins/enzymes. The dethiolation activity is NADPH-dependent and requires TR to catalyze the conversion of oxidized TRx to its reduced state. TRx was first isolated as a hydrogen donor for the enzymatic synthesis of deoxyribonucleotides by ribonucleotide reductase in *E. coli* [81]. Recent studies suggest that TRx plays a variety of roles in transcription, growth control, and immune function [82,83]. *TRx* gene expression is essential for early differentiation and morphogenesis of embryos; thus, the deletion of this gene is lethal [84].

**Figure 8:**
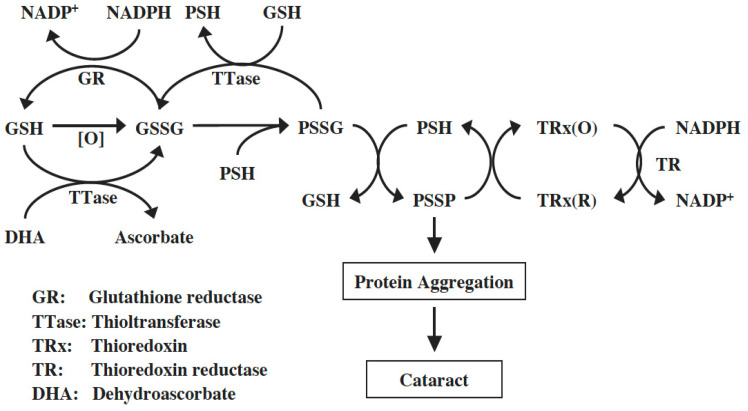
Thiol regulation in the lens. Reprinted from Lou [72] with permission from Pergamon.

Both *TRx-1* and *TRx-2* genes are expressed in human and mouse lenses [85,86]. The human lens *TRx-1* gene was cloned, and the purified recombinant protein was confirmed to be identical to TRx from other human tissues [87]. TRx-1 and TR were present in human lens epithelial cells at a higher level than in other regions of the lens. Furthermore, cultured pig lenses pre-exposed to H_2_O_2_ showed a transient and quick upregulation of TRx, TR, and TTase-1 prior to cataract induction, likely a process used by the lens to combat oxidative stress [87]. Therefore, the TRx/TR system is functional in the lens and, along with the GSH/TTase system, may synergistically regulate redox homeostasis in the cells as proposed in the diagram below (Figure 8). For more detailed information please read the review by Lou [72].

## 9. Loss of Thiol Repair Systems in Aging and Cataractous Lenses

With the above evidence showing how important TTase is in maintaining SH/-S-S- balance and protecting lens proteins/enzymes from oxidation, one could predict that age-related cataract formation may be partly due to weakened or decreased thiol-regulating systems with aging. It also seems likely that a cataractous lens may have suboptimal levels of TTase and TRx.

### 9.1. Thiol Repair Systems in Normal Mouse and Human Lens as a Function of Age

**Figure 9:**
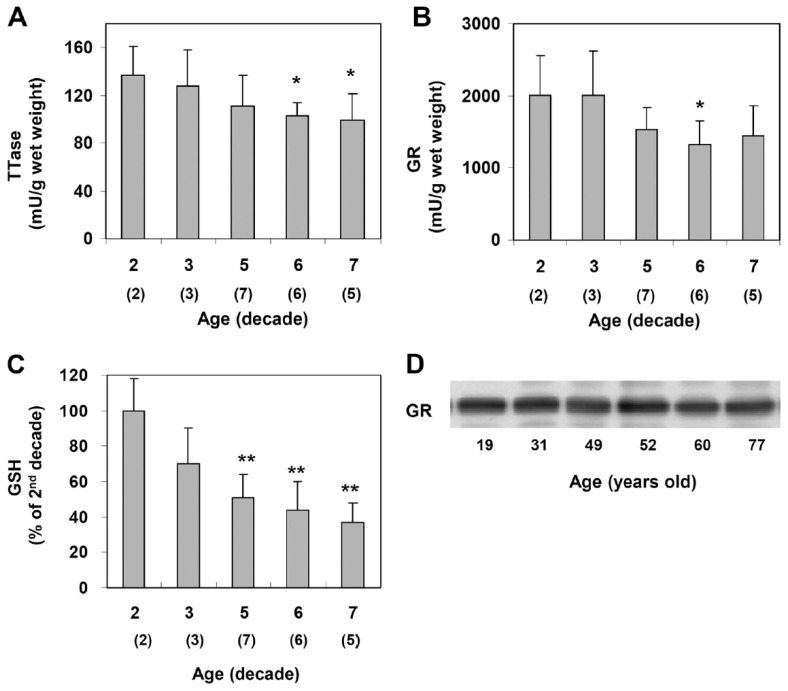
Age-dependent changes in the TTase system in the human lens. Twenty-three normal human lenses, divided into second, third, fifth, sixth, and seventh decades, were used for the study. The data are shown as mean ± SD, with the number of lenses indicated as (n) in each decade. (**A**). TTase activity in mU/g lens wet weight. (**B**). Glutathione reductase (GR) activity in mU/g lens wet weight. (**C**). GSH level expressed as a percentage of the second decade. (**D**). Homogenates from 19-, 31-, 49-, 52-, 60- and 77-year-old lenses were selected for immunoblot analysis for GR with a specific anti-GR antibody. The blot shown is a representative of three separate analyses. * *p* < 0.05 vs. 2nd decade; ** *p* < 0.005 vs. 2nd decade. Reprinted from Xing and Lou [88] @ ARVO.

Xing and Lou [88] examined normal human lenses from decades 2, 4, 5, 6, and 7 and confirmed the findings of age-dependent GSH loss in the literature [12,27]. However, the activities of TTase and glutathione reductase (GR) in the thiol repair systems were also progressively decreased with age (Figure 9), suggesting that the less active repair systems may lead to the accumulation of thiolated proteins and protein aggregates during aging. Furthermore, the second thiol regulating system of TRx/TR was also progressively decreased with age [88]. Therefore, as expected, aging is definitely a risk factor in thiol repair protection and, thus, is a higher risk in oxidant-induced cataract formation.

The mouse lens also displayed definite age-dependent differences between 1- and 16-month-old animals [89]. For instance, older lenses at 16 months showed a 20% loss in the GSH pool while thiolated total lens proteins increased 30–40%, as detected by Western blot with anti-GSH antibody. Key enzymes were severely inactivated during aging with a nearly 70% loss in glyceraldehyde 3-phosphate dehydrogenase (G-3PD) activity, a key glycolysis enzyme, and about a 60% drop in TTase activity and expression. As expected, all 16-month-old mice showed cortical lens opacity.

An attempt was made to investigate if the aging lens with weaker activity or lower levels of TTase will influence UV-induced cataract formation. The results of Zhang et al. [89] confirmed their speculation that the 16-month-old in comparison to the 1-month-old showed much faster and more severe development of lens opacity in superficial anterior subcapsular cataracts. The biochemical alterations in these old lenses were more prominent in the UV-exposed older animals, such as the high elevation of PSSG, the loss in GSH, and decreased protein solubility. Interestingly, when the authors used a protocol to examine the potential recovery in one-day UV-treated animals, only the young animals had the ability to restore lens transparency with normalized GSH and PSSG levels beginning at day 1 and completely by day 8 [89]. As was observed in the early stages of H_2_O_2_-exposed human lens epithelial cells [90] and intact pig lenses [91], TTase and TRx were transiently upregulated 2–3-fold under these experimental conditions [91]. Therefore, it is quite reasonable to speculate that upregulation or increased TTase and TRx activities helped the young lens achieve its ability to balance SH/S-S homeostasis in the lens with restored protein functions and enzyme activities.

### 9.2. Thiol Repair Systems in Cataractous Lenses

**Figure 10:**
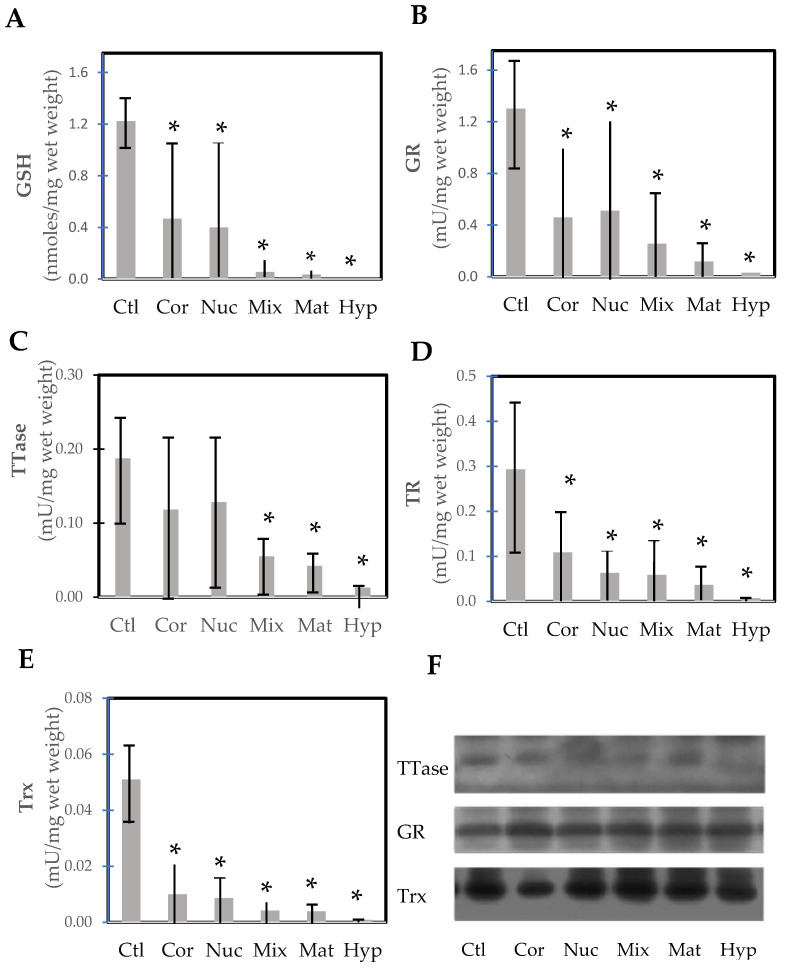
Thiol repairs enzyme activities and GSH levels in the nucleus of normal control and various types of cataractous lenses (cortical, nuclear, mixed cataract, mature, and hypermature cataracts). (**A**) GSH; (**B**) glutathione reductase (GR); (**C**) thioltransferase (TTase); (**D**) thioredoxin reductase (TR); (**E**) thioredoxin (Trx); (**F**) immunoblot analysis of TTase, GR, and Trx. * *p* < 0.05 compared to control. Replotted from Wei et al. [92].

Wei et al. [92] conducted a rare but important study examining lens opacity in relation to the thiol protecting and repairing enzymes in extracapsular cataract extraction (ECCE) human cataractous lenses. Several cataract surgeons were involved in this study by first grading and then collecting the lenses via the ECCE procedure on-site with patients 57–85 years of age in the mountainous region in China, sponsored by an international project. A large number of categorized cataract lenses could be examined, including cortical, nuclear, mixed, mature, and hypermature cataracts. In comparison to the nuclear region of normal lenses from 49–71-year-olds, the thiol protection system of GSH/GR and the repair systems of TTase and TRx in these tissues were progressively diminished by 70–90% to a near total loss, depending on the degree of opacity from cortical to hypermature cataract (Figure 10). Similarly, ATP level and G-3PD activity were also extensively depleted in these lenses. One interesting finding in this study was that the protein levels of TRx, TTase, and GR all remained at the same level as those of the control lenses. This indicates that the activity loss in the cataractous lenses was due to post-translational modification of the enzymes but not from a decrease in capacity for gene expression. This finding is in agreement with the earlier report [72,93], and the hypothesis proposed by Lou et al. [20] that the formation of age-related cataracts may be initiated in part by the oxidation-induced protein modification of thiol groups leading to protein aggregation and insolubility.

Post-translational modification may also offer an explanation for the formation of methionine sulfoxide (MetSO) in cataracts. The enzyme methionine sulfoxide reductase, which can reduce protein-bound MetSO back to Met [94,95], has been found in the lens [96]. Activity is found in both the nucleus and cortex, with very little difference between them. It has been suggested that the enzyme offers protection from oxidation [97,98]. However, the enzyme specifically requires thioredoxin (TRx) for its action. As found by Wei et al. [92] TRx in the normal lens cortex and nucleus as well as in various cataracts is inactive (Figure 10E) but the amount of protein is constant, suggesting post-translational modification may have rendered the enzyme inoperative. This is an area that requires further investigation.

Several additional interesting findings in the above studies are worth noting. (1). The oxidation damage protection system appears to link to the clinical aspect of vision. GSH and the thiol damage repair enzyme systems were only partially depleted or inactivated when the patients still had some good vision but not in those patients who had very poor vision. (2). Most of the activities of the redox–regulating enzymes were suppressed more in a lens with opacity in the cortex than in the nucleus. Thus oxidation-related damage in the nucleus may not be as obvious as that of the cortical region. (3). More extensive diminished G-3PD and ATP pools were noted in both the cortical and nuclear regions in a cortical cataract than in a nuclear cataract.

## 10. Effect of TTase Deletions on Cell Function and Lens Transparency

To further understand the role of TTase in regulating SH/S-S in the lens, the Lou laboratory acquired a TTase-1 gene knockout (KO-1) mouse line from Dr. Ho of Wayne State University in which the TTase gene was knockout by deleting exon 2, the GSH binding site. This mouse line lacks TTase-1 in many organs, including the brain, heart, kidney, liver, lung, and lens.

**Figure 11:**
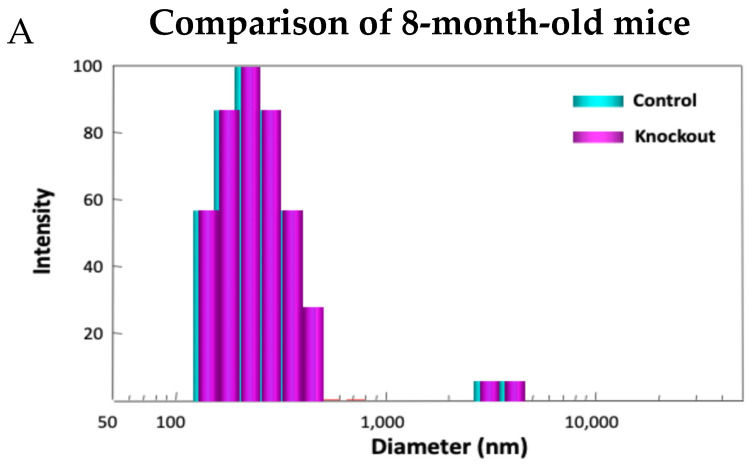

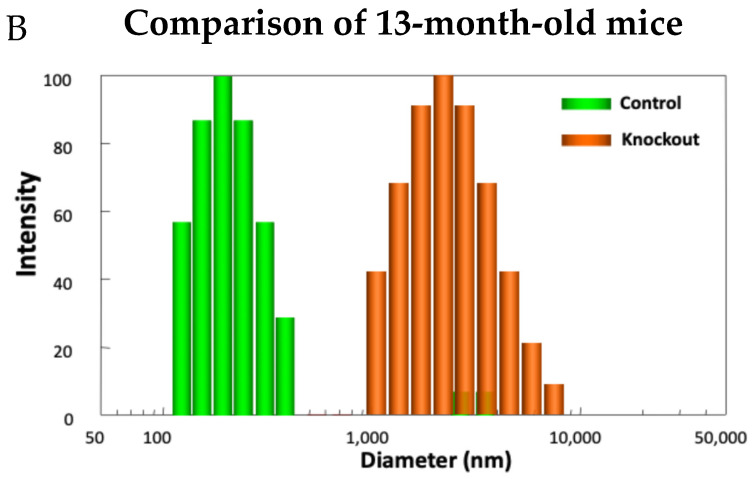
Distribution of HMW particles in 8-month-old (**A**) and 13-month-old (**B**) control and TTase KO mice, measured by dynamic light scattering (DLS).

Examining the model in detail revealed how lacking TTase-1 could adversely affect the well-being of the animal and its lens [99]. First, the KO-1 mouse showed slower growth both in body weight and in lens weight. Second, the lenses from 2-month-old KO-1 mice in organ culture showed lower resistance to oxidative stress and formed opacity more easily in H_2_O_2_-containing medium compared to lenses from wild-type mice of the same age. Third, lacking TTase caused protein aggregation in an intact lens. In collaboration with Dr. Ansari’s NASA team, laser dynamic light scattering (DLS) was used to detect high molecular weight (HMW) particles in the lenses of wild-type and KO-1 mice. Similar small particles of 100–700 nm diameter were found in the 8-month-old lenses from both wild-type (control) and KO mice. A remarkable increase in HMW particles (1000–10,000 nm diameter) both in size and quantity was found in 13-month-old KO-1 mice but none in the age-matched WT control (Figure 11). Furthermore, the presence of HMW was age-dependent. When the age increased from 3–8 months to 18–25 months, HMW particles showed a limited increase in the WT but a drastic elevation both in size and abundance in the KO-1 mice.

**Figure 12:**
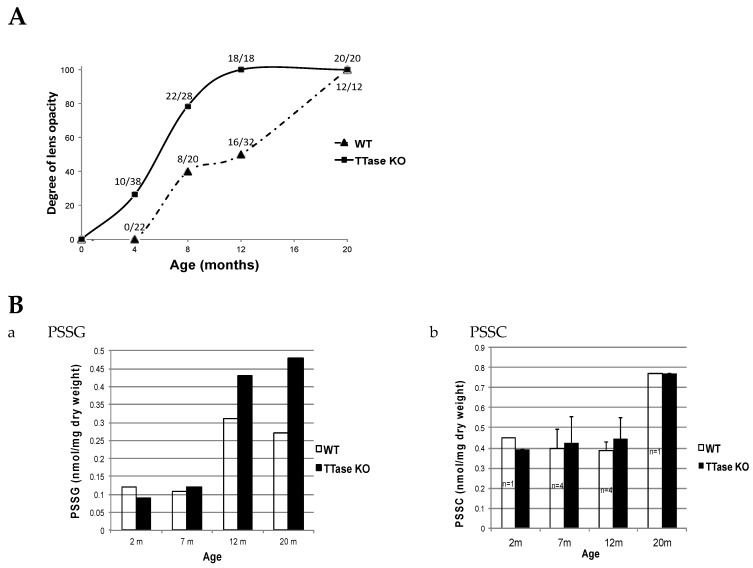
The effect of TTase deletion in lens opacity and the level of protein–thiol mixed disulfides in WT and TTase KO mice. (**A**). The percentage of cataract eyes detected in WT and KO-1 mice colonies during 4–20 months. (**B**). A comparison of the protein–thiol mixed disulfides, PSSG (**a**) and PSSC (**b**), during aging (2–20 m).

The possible effect of TTase deletion on the SH/S-S balance and age-related cataract formation was also examined using the same model in vivo. Zhang et al. [100] used TTase KO and WT mice of matching age between 1 and 25 months to monitor lens clarity with a slit lamp, and classified and graded the lens opacity according to the LOSII system [101]. Free GSH and protein–thiol mixed disulfides were monitored in the same animal lenses for age-related changes. It was clearly shown that lens opacity appeared much earlier and was denser in the KO mice. When monitoring the progression of lens opacity during aging in a large population of WT and KO-1 mice, about one-quarter of the 4-month-old KO mice showed lens opacity. By 8 months, over half had developed opacity, while all had cataracts in both the 12- and 22-month groups. On the other hand, lens opacities in WT controls only began to appear at 8 months. Half of them had cataracts by 12 months and nearly all lenses showed opacity by 22 months (Figure 12A). For the changes of PSSG levels during aging (Figure 12B), the TTase KO mice levels were progressively higher than those of the wild-type control at 7–20 months, while the change of PSSC was relatively minor with a minimal difference between the KO and the WT mice.

The above results with the TTase KO mouse model strongly support the importance of this redox-regulating enzyme in protecting the lens from oxidative stress and cataract formation. Although the PSSG level in the lens of KO mice was not extensively elevated during the young age when opacity began to appear, it is important to point out that the fact that the PSSG formed, even at a low level, but without TTase regulation, could be enough to alter protein conformation. The subsequent impairment of protein/enzyme functions would eventually affect lens transparency. Finally, the findings [102,103] that the TTase KO mouse was more susceptible to UV radiation-induced cataracts, whereas enhanced TTase gene levels provided protection, provide additional evidence showing how important TTase is in protecting the lens from oxidant-induced cataract formation. Interestingly, recent studies [104,105] have also shown that the absence of TTase-1 or Grx1 affected a more aggravated epithelial–mesenchymal transition, resulting in posterior opacification (PCO) after cataract surgery.

## 11. Conclusions

Based on the above animal and human cataract studies, one may conclude the following:

(1). Oxidative stress is directly involved in many types of cataract formation through oxidation of GSH. (2). Glutathionylation of the lens proteins (PSSG) is a major player in causing protein conformation changes, resulting in protein–protein disulfide formation, insolubility, and protein aggregation. (3). The thiol/disulfide-regulating enzyme systems of thioltransferase and thioredoxin/thioredoxin reductase are present in the lens to control and repair glutathionylation-initiated damages. (4). Senile cataract formation is mainly due to loss of the lens thiol/disulfide-regulating systems during aging. (5). TTase knockout mice may be a good model for studying the mechanism of senile cataract formation, and TTase gene enhancement may be a potential therapeutic tool for anticataract treatment.

For years, it has been debated whether PSSG accumulation in the lens is the end result of cataract formation or the cause of cataracts. It is reasonable to argue that both hypotheses have merit since they are essentially the two ends of the same situation. Only a small amount of PSSG may be needed initially to trigger the cascading damage effects to the lens proteins. On the other hand, the events leading to lens opacity would certainly cause accumulation of PSSG in high quantity if TTase is not active or present in an adequate amount to dethiolate the glutathionylated proteins to repair the damage. Thiolation of certain proteins in the lens could be a protective event. If this is the possible function of thiolation, it may be effective initially when an active TTase is available to remove the GS- or CS-adduct and restore the protein-SH in time. However, if the adduct is not removed in time, the same protein would be affected by the extra charge and weight of GS- or CS-adducts to change its conformation, followed by some irreversible damage. All in all, the key to a healthy and transparent lens is that it must have its thiol/disulfide regulation system intact and in place. This situation is, undoubtably, not achievable in an aging lens, thus making it highly vulnerable to cataract formation.

The changes observed with whole lens model studies appear to be consistent with cortical cataract formation. However, how it applies to a nuclear cataract, particularly nuclear cataract in humans, is not certain as the formation of fluorescence, intense pigmentation, and non-disulfide covalent crosslinking are hallmarks of a typical human nuclear cataract. Whether protein glutathionylation plays a part in such cataract formation needs to be further investigated. In addition, what is the role of PSSC? Its unique presence and elevation in the cataracts involving the nuclear region is an intriguing observation. These and even the simple questions of whether the mechanism of cataract formation in cortical cataracts differs from nuclear cataracts are all interesting topics that need more research attention in the future.
